# Acute Retinal Pigment Epitheliitis: Spectral Domain Optical Coherence Tomography, Fluorescein Angiography, and Autofluorescence Findings

**DOI:** 10.1155/2015/149497

**Published:** 2015-02-12

**Authors:** Tuğba Aydoğan, Esra Güney, Betül İlkay Sezgin Akçay, Tahir Kansu Bozkurt, Cihan Ünlü, Ahmet Ergin

**Affiliations:** Eye Clinic, Umraniye Training and Research Hospital, Umraniye, 34766 Istanbul, Turkey

## Abstract

A 17-year-old presented with central and paracentral scotomas in his right eye for one week. There was no remarkable medical or ocular history. Blood analyses were within normal range. At presentation both eyes' best-corrected visual acuities were 20/20. Slit-lamp examination result was normal. Fundus examination revealed yellow-white hypopigmented areas in the macula. Fluorescein angiography (FA) showed hypofluorescence surrounded by ring of hyperfluorescence. Fundus autofluorescence (FAF) was slightly increased. Spectral domain optical coherence tomography (SD-OCT) showed disruption of IS/OS junction with expansion of abnormal hyperreflectivity from retinal pigment epithelium to the outer nuclear layer (ONL). One month later fundus examination showed disappearance of the lesions. FA revealed transmission hyperfluorescence. FAF showed increased autofluorescence and pigment clumping. Hyperreflective band in SD-OCT disappeared. Loss of photoreceptor segment layers was observed in some of the macular lesions. The diagnosis of acute retinal pigment epitheliitis can be challenging after disappearance of fundus findings. FA, FAF, and SD-OCT are important tests for diagnosis after resolution of the disease.

## 1. Introduction

Acute retinal pigment epitheliitis (ARPE), which is first described by Krill and Deutman [1], is a self-limiting disease of unknown aetiology typically affecting healthy young adults. The disease is characterized by acute onset of visual symptoms (blurred vision, central/paracentral scotomas, and metamorphopsia) which resolve without treatment within 6 to 12 weeks [1–4].

The diagnosis usually depends on symptoms and fundus examination findings. Spectral domain optical coherence tomography (SD-OCT) and fluorescein angiography (FA) are ancillary tests which give us an understanding for the exact spatial location of the disrupted retinal layer. Fundus findings include small areas of fine pigment stippling surrounded by hypopigmented yellow-white haloes in the macular region [1–9]. OCT reveals disruption of the photoreceptors inner segment and outer segment (IS-OS) junction in association with a hyperreflective band [6–11]. The hypopigmented areas show transmission hyperfluorescence without leakage on FA [2, 4, 6–9, 12].

Herein we report SD-OCT, FA, and fundus autofluorescence (FAF) findings of a patient diagnosed with ARPE.

## 2. Case Report

**Figure 1:**
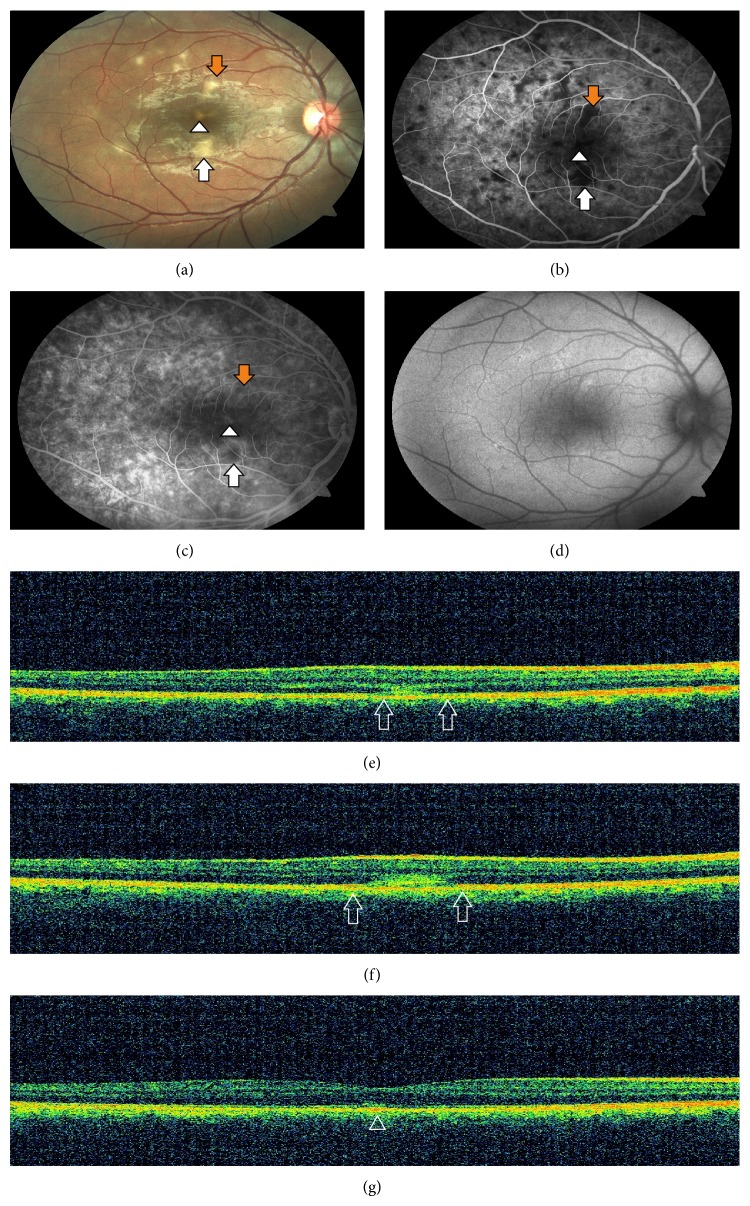
(a) Fundus photography demonstrating vertically aligned yellow-white hypopigmented areas involving superior (orange arrow), inferior (white arrow), and central foveal regions (arrow head) and other hypopigmented areas superotemporally. (b) Early phase of the FA showing hypofluorescence corresponding to hypopigmented areas that were surrounded by a ring of hyperfluorescence. (c) Late phase of the FA showing increased hyperfluorescence due to staining. (d) FAF showing slightly increased autofluorescence in the corresponding hypopigmented areas. (e)-(f) SD-OCT showing disruption of IS/OS junction with expansion of abnormal hyperreflectivity from retina pigment epithelium (RPE) to the outer nuclear layer (ONL) in the superior and inferior regions (both figures, between the two arrows). (g) SD-OCT showing RPE irregularities with minimal disruption of IS/OS junction in the foveolar lesion (arrowhead).

A 17-year-old man presented with a complaint of central and paracentral scotomas in his right eye for 1 week. There was no remarkable medical or ocular history. Blood analyses were within normal range. Slit-lamp examination result was normal. Best-corrected visual acuity was 20/20. Fundus examination revealed vertically aligned yellow-white hypopigmented areas involving superior, inferior, and central macular regions. Other hypopigmented areas were noticed superotemporally. Fine pigment stippling was detected only in the foveola (Figure 1(a)). No sign of vitritis was seen. Early phase of the FA showed hypofluorescence corresponding to hypopigmented areas that were surrounded by a ring of hyperfluorescence (Figure 1(b)). Hyperfluorescence increased due to staining at the late phase of the FA (Figure 1(c)). FAF was slightly increased in the hypopigmented areas (Figure 1(d)). SD-OCT showed disruption of IS/OS junction with expansion of abnormal hyperreflectivity from RPE to the outer nuclear layer (ONL) in superior and inferior lesions (Figures 1(e) and 1(f)). Inferior lesion which was larger showed inner RPE disruption. Foveolar lesion showed RPE irregularities with minimal disruption of IS/OS junction (Figure 1(g)).

**Figure 2:**
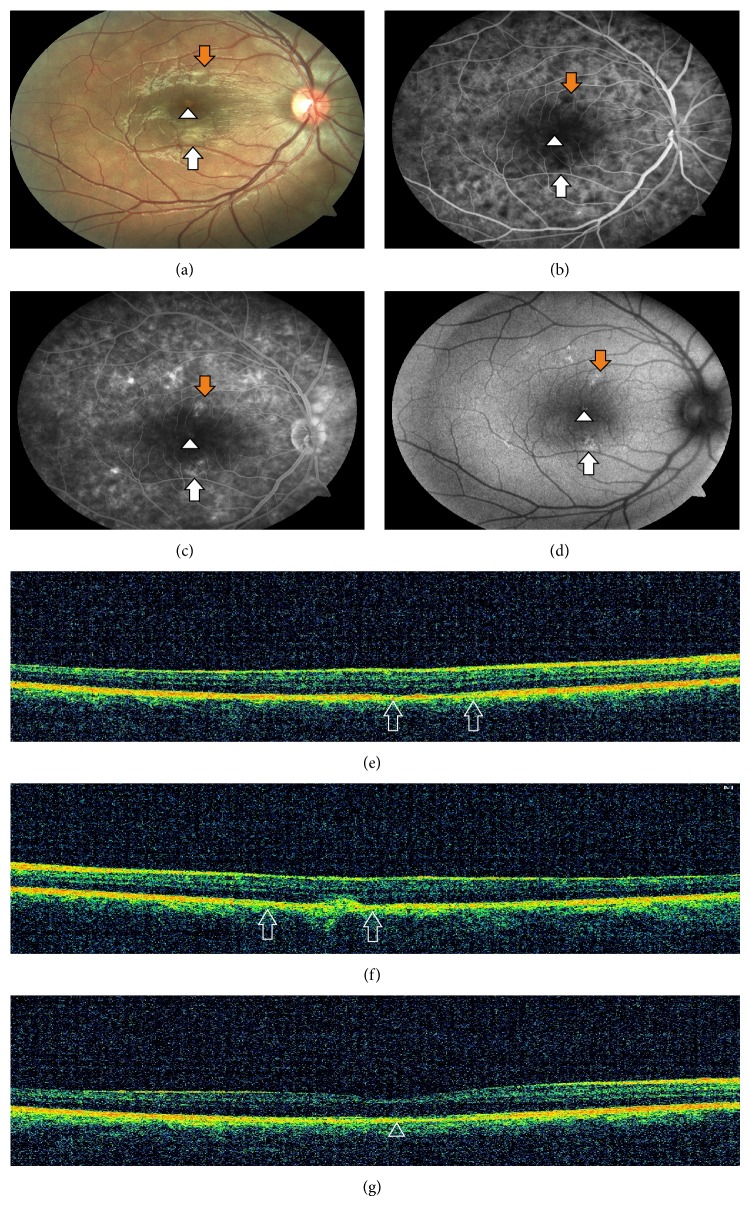
(a) Fundus photography demonstrating slightly disappeared hypopigmented spots one week later. (b) Early phase of the FA showing increased hyperfluorescence surrounding hypofluorescent areas compared to initial FA. (c) Late phase of the FA showing further increase in hyperfluorescence due to staining. (d) FAF showing increased autofluorescence and pigment clumping in the corresponding hypopigmented areas. (e) SD-OCT showing the disappearance of the hyperreflective band, loss of photoreceptor segment layers, and RPE irregularities in the superior lesion (between the two arrows). (f) SD-OCT showing the hyperreflective band in the inferior lesion, which became smaller in size and loss of photoreceptor segment layers (between the two arrows). (g) SD-OCT showing RPE irregularities with minimal disruption of IS/OS junction in the foveolar lesion (arrowhead).

One week later subtle improvement was observed in visual symptoms. Hypopigmented areas slightly disappeared (Figure 2(a)). FA showed increased hyperfluorescence surrounding the hypofluorescent areas compared to initial FA (Figure 2(b)). Late phase of the FA showed further increase in hyperfluorescence due to late staining (Figure 2(c)). FAF showed increased autofluorescence and pigment clumping (Figure 2(d)). SD-OCT demonstrated disappearance of the hyperreflective band involving the ONL and the photoreceptor segment layers, loss of photoreceptor segment layers, and RPE irregularities in the superior lesion (Figure 2(e)). The hyperreflective band persisted but became smaller in size in the inferior lesion (Figure 2(f)). Foveolar lesion remained the same with scarce RPE irregularities (Figure 2(g)).

**Figure 3:**
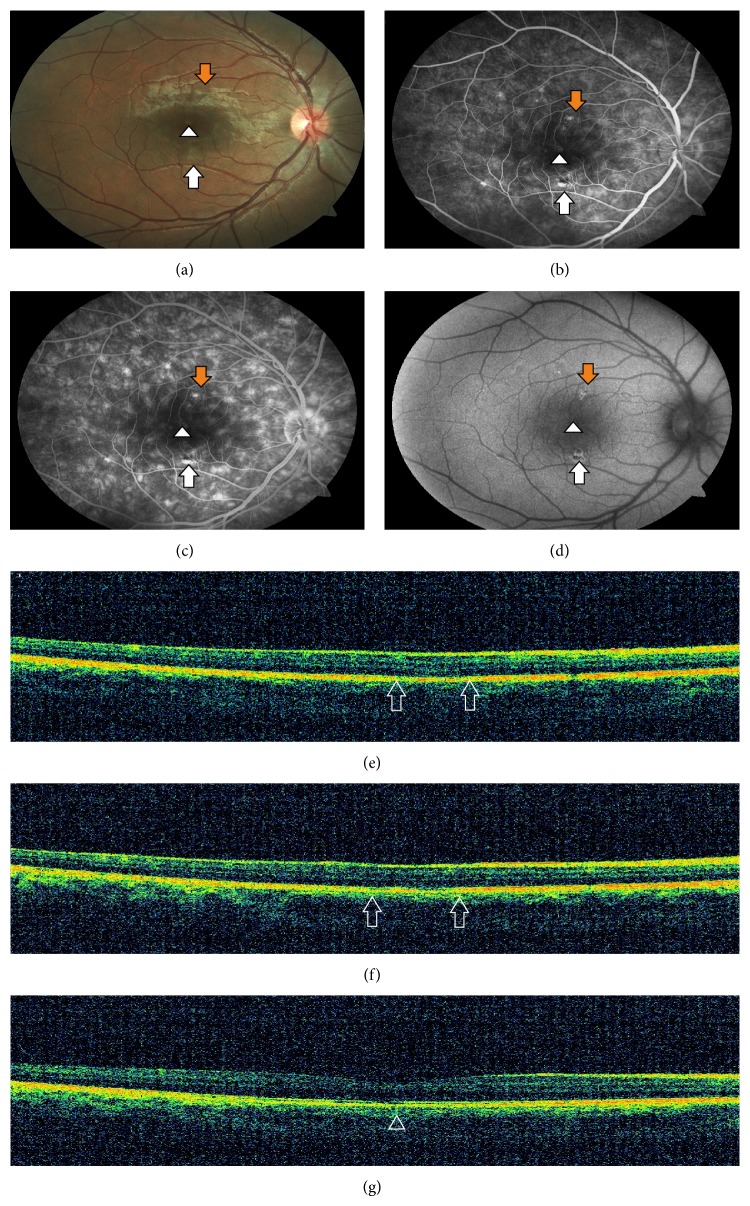
(a) Fundus photography showing almost disappeared lesions only with a slight residual hypopigmentation. (b) Early phase of the FA demonstrating more prominent transmission hyperfluorescence compared to previous FAs in the affected areas. (c) Late phase FA demonstrating slightly increased hyperfluorescence due to staining. (d) FAF showing increased autofluorescence and more pigment clumping in the corresponding hypopigmented areas. (e)-(f) SD-OCT showing loss of photoreceptor segment layers with slightly irregular RPE layers in the superior and the inferior lesions (both figures, between the two arrows). (g) SD-OCT showing decreased RPE irregularities and restored IS/OS junction in the foveolar lesion (arrowhead).

One month later visual symptoms recovered. In the fundus examination lesions disappeared leaving behind slight residual hypopigmentation (Figure 3(a)). Beginning from early phase of the FA more prominent transmission hyperfluorescence was seen in the affected areas compared to previous FAs (Figure 3(b)). At the late phase FA, hyperfluorescence slightly increased due to late staining (Figure 3(c)). Pigment clumping and autofluorescence significantly increased in FAF (Figure 3(d)). Hyperreflective bands disappeared in all of the lesions. In the superior and inferior lesions loss of photoreceptor segment layers was observed with slightly irregular RPE (Figures 3(e) and 3(f)). RPE irregularities recovered and IS/OS junction was observed to be restored in the foveolar lesion (Figure 3(g)).

## 3. Discussion

ARPE is an acute, self-limiting disease of unknown aetiology. It typically affects healthy young adults. The pathogenesis and aetiology are still unclear. Although there were reports about FA and SD-OCT characteristics of the lesions, from the time Krill and Deutman described the disease entity, only a few case reports were published [2–10, 12, 13].

Chittum and Kalina [2] described FA findings as transmission hyperfluorescence without leakage seen in the hypopigmented areas. Several reports also showed transmission hyperfluorescence in the FA [2, 4, 6–9, 12]. Our FA findings changed over time. FA initially showed early hypofluorescent areas later stained in a halo pattern. Eventually FA findings became more prominent and beginning from the early phase of the fluorescein angiography transmission hyperfluorescence without leakage was observed in the corresponding hypopigmented areas. FA findings can be variable at different stages of the disease. Recognition of different FA findings is important for accurate diagnosis.

Hsu et al. [6] was first to describe the SD-OCT findings and reported abnormal increased reflectivity involving the ONL and the RPE with absence of intraretinal, subretinal, or sub-RPE fluid. Our patient's SD-OCT findings included disruption of IS/OS junction with expansion of abnormal hyperreflectivity from RPE to the ONL especially in the superior and inferior lesions. Hypopigmented lesion in the fovea showed a slight disruption of IS/OS junction with mild RPE irregularities. The lesion in the fovea almost fully recovered in a month when compared to other lesions in which loss of photoreceptor layer was seen after resolution of the hyperreflective bands. This finding could be the result of varied degrees of involvement in different areas of macula.

There are only a few reports mentioning FAF. In some reports autofluorescence measurement was normal [7, 10]. Increased autofluorescence with pigmented dots was also detected in corresponding areas [12]. In our case fundus autofluorescence findings altered during follow-up. Initially slightly increased autofluorescence was detected. After disappearance of the lesion in the fundus examination, FAF showed increased autofluorescence and pigment clumping. Changes seen in RPE layer are usually permanent in ARPE [2]. FAF is an important tool for diagnosis even after disease recovery.

Increased autofluorescence and pigment clumping were detected in the areas of photoreceptor layer loss and RPE changes. Both SD-OCT and fundus autofluorescence findings showed us involvement of ONL has worse prognosis causing loss of photoreceptor layer when compared to slight disruption of IS/OS junction and RPE irregularities which recovered almost completely.

The pathogenesis of the disease is still unclear. Hsu et al. [6] hypothesized that primary inflammation could involve photoreceptors causing a secondary subacute response in the RPE. Recently Cho et al. [11] suggested that the initial lesion in acute retinal pigment epitheliitis located at the junction between the photoreceptor outer segments and the apical sides of the RPE cells. In our patient the more severely affected lesions involved the outer nuclear layer, photoreceptor segment layer, and RPE inner layer. Less severe lesion caused RPE irregularities and only a slight disruption of IS/OS junction. In the light of this finding it can be suggested that initial lesion is located at the RPE or junction between the RPE and the photoreceptor outer segments.

ARPE can be recognized with FA, FAF, and SD-OCT findings even after the lesions seen in fundus examination were resolved. Recognizing the disease is important to avoid unnecessary treatment.
